# Consolidated bioprocessing of corn cob-derived hemicellulose: engineered industrial *Saccharomyces cerevisiae* as efficient whole cell biocatalysts

**DOI:** 10.1186/s13068-020-01780-2

**Published:** 2020-08-08

**Authors:** Joana T. Cunha, Aloia Romaní, Kentaro Inokuma, Björn Johansson, Tomohisa Hasunuma, Akihiko Kondo, Lucília Domingues

**Affiliations:** 1 CEB–Centre of Biological Engineering, University of Minho, Campus Gualtar, Braga, Portugal; 2 Graduate School of Science, Technology and Innovation, Kobe University, 1-1 Rokkodai-cho, Nada-ku, Kobe, Hyogo 657-8501 Japan; 3 Center of Molecular and Environmental Biology (CBMA), University of Minho, Braga, Portugal; 4 Engineering Biology Research Center, Kobe University, 1-1 Rokkodai-cho, Nada-ku, Kobe, Hyogo 657-8501 Japan

**Keywords:** Consolidated bioprocessing, Cell-surface display, Lignocellulosic ethanol, Industrial *Saccharomyces cerevisiae*

## Abstract

**Background:**

Consolidated bioprocessing, which combines saccharolytic and fermentative abilities in a single microorganism, is receiving increased attention to decrease environmental and economic costs in lignocellulosic biorefineries. Nevertheless, the economic viability of lignocellulosic ethanol is also dependent of an efficient utilization of the hemicellulosic fraction, which contains xylose as a major component in concentrations that can reach up to 40% of the total biomass in hardwoods and agricultural residues. This major bottleneck is mainly due to the necessity of chemical/enzymatic treatments to hydrolyze hemicellulose into fermentable sugars and to the fact that xylose is not readily consumed by *Saccharomyces cerevisiae*—the most used organism for large-scale ethanol production. In this work, industrial *S. cerevisiae* strains, presenting robust traits such as thermotolerance and improved resistance to inhibitors, were evaluated as hosts for the cell-surface display of hemicellulolytic enzymes and optimized xylose assimilation, aiming at the development of whole-cell biocatalysts for consolidated bioprocessing of corn cob-derived hemicellulose.

**Results:**

These modifications allowed the direct production of ethanol from non-detoxified hemicellulosic liquor obtained by hydrothermal pretreatment of corn cob, reaching an ethanol titer of 11.1 g/L corresponding to a yield of 0.328 g/g of potential xylose and glucose, without the need for external hydrolytic catalysts. Also, consolidated bioprocessing of pretreated corn cob was found to be more efficient for hemicellulosic ethanol production than simultaneous saccharification and fermentation with addition of commercial hemicellulases.

**Conclusions:**

These results show the potential of industrial *S. cerevisiae* strains for the design of whole-cell biocatalysts and paves the way for the development of more efficient consolidated bioprocesses for lignocellulosic biomass valorization, further decreasing environmental and economic costs.

## Background

Lignocellulosic biomass is a sustainable resource for the production of biofuels, and its utilization is a possible solution to alleviate the current world dependence on fossil fuels. In this context, consolidated bioprocessing (CBP), in which the same microorganism is able to produce hydrolytic enzymes and ferment sugars into ethanol [1], emerges as a promising alternative configuration to simultaneous saccharification and fermentation (SSF) process [2] and holds great promise for the efficient conversion and valorization of lignocellulosic biomass. Enzymes can be either secreted or displayed on the cell surface. The cell-surface display strategy exhibits several advantages, such as: (i) high localized enzyme activity, (ii) the monosaccharide release occurs close to the cell surface being instantaneously consumed by the cell, which reduces the risk of contamination or product inhibition, (iii) the immobilization of enzymes on cell surface allows their re-utilization in successive cultures, which lowers the overall process cost [3]. Moreover, this strategy enables the use of recombinant microorganisms as whole-cell biocatalysts. The whole-cell biocatalysis provides a sustainable alternative to traditional chemical catalysis, since biocatalysis presents higher selectivity and catalytic efficiency, can be carried out at milder operation conditions and multi-step reactions can be performed in a single strain allowing cofactor regeneration [4]. Moreover, the CBP strategy could contribute to a lower bioethanol production cost from lignocellulosic biomass, since CBP combines three process stages in a single recombinant microorganism, namely: the enzyme production, enzymatic saccharification and sugar fermentation. The effective use of hemicellulose is fundamental for bioethanol production as xylan may be a major constituent of some lignocellulosic biomasses, e.g., corn cob can contain up to 31 g of xylan per 100 g of raw material [5]. Xylan from the hemicellulosic fraction of agro-industrial residues is an amorphous heteropolymer that comprises a backbone of β-1,4-linked xylose partially substituted with acetyl groups, uronic acids and arabinose [6]. The removal of the xylan side chains requires different enzymes dependent of the substituent (e.g., acetylxylan esterases for acetyl, α-l-arabinofuranosidases for arabinose), while degradation of the xylan backbone into xylose monomers requires *endo*-1,4-β-xylanases and β-1,4-xylosidases: the main chain of xylan can be hydrolysed into xylooligosaccharides (XOS) by *endo*-1,4-β-xylanases, which can be cleaved into xylose by β-1,4-xylosidases [7]. Hydrothermal treatment of lignocellulosic biomass is a recognized environmental friendly process that improves the enzymatic saccharification of cellulose promoting the solubilization of hemicellulose as hemicellulosic-derived compounds (mainly composed by XOS). Still, these XOS are not metabolized by the yeast *Saccharomyces cerevisiae*, main microbial ethanol producer. Thus, the direct conversion of hemicellulose into ethanol requires the simultaneous expression of xylan-degrading enzymes and xylose-assimilating enzymes in the recombinant S*. cerevisiae* strain. Despite promising advantages of CBP process over other alternatives, few attempts have been reported for the direct conversion of hemicellulose into ethanol, achieving ethanol concentrations in the range from 0.32 to 8.2 g/L [6, 8–12]. The most remarkable results with cell-surface display in a hemicellulosic CBP were obtained with liquors derived from hydrothermal treatment of rice straw, in which 4.04 and 8.2 g/L of ethanol were produced by engineered industrial *S. cerevisiae* Sun049 strain and engineered laboratorial *S. cerevisiae* NBRC1440/X strain, respectively [6, 8].

One of the most important limitation of CBP process is the selection of operating temperature, since ethanologenic yeast (such as *Saccharomyces cerevisiae*) and xylanases/cellulases work at different range of temperatures. In addition, the engineered yeast has to function under adverse conditions, such as the presence of inhibitors derived from pretreatment (including acetic acid, furfural and 5-hydroxymethylfurfural (HMF)). In this sense, the selection of host microorganism with desired features such as thermotolerance and resistance to inhibitors is crucial to develop this sustainable bioprocess. Previous works have demonstrated the specific robustness [13, 14] and superior capacity of yeast strains isolated from industrial environments for lignocellulosic fermentation in these demanding conditions [15]. In addition, hemicellulosic CBP requires a highly engineered yeast, as xylose consumption pathways must be expressed together with xylan degrading enzymes. In this context, recent works have indicated that industrial isolates can present different intrinsic abilities to cope with genetic engineering strategies for xylose consumption [16] and expression of hydrolases [17, 18]. Therefore, there is a need for a tailor-made development of hemicellulosic ethanol-producing yeast, where intrinsic capabilities of host microorganism (suitable for the process) are previously selected.

Taking into account the interest of the development of CBP as a green alternative to chemical catalysis in a lignocellulose biorefinery context, the aim of this study was to (1) explore the potential of industrial *S. cerevisiae* strains as host for the design of whole-cell biocatalyst through cell-surface display of hemicellulose-degrading enzymes together with the expression of xylose consumption pathways, (2) evaluate their performance in non-detoxified corn cob liquor in terms of hemicellulose saccharification and direct conversion into ethanol, and (3) compare the efficiency of CBP against a SSF process with addition of commercial hemicellulases.

## Results and discussion

### Corn cob processing: hydrothermal treatment for hemicellulosic liquors

To evaluate the capacity of the constructed strains for the enzymatic saccharification and fermentation of hemicellulose, corn cob was selected as a representative renewable resource due to its high xylan content. The chemical composition of corn cob (expressed in g/100 g of raw material in oven-dry ± standard deviation based on three replicate determinations) was as follows: 28.79 ± 1.45 of glucan, 29.63 ± 0.45 of xylan, 3.62 ± 0.13 of arabinan, 2.58 ± 0.05 of acetyl groups and 18.58 ± 0.87 of Klason lignin. Corn cob was submitted to hydrothermal treatment under conditions selected based on previous works [5, 19]. Table 1 shows the chemical composition of solid and liquid phases after pretreatment at different severities (*S*_0_, 3.67, 3.99 and 3.78, corresponding to *T*_max_ of 205, 211 and 207 °C in non-isothermal regime) and liquid–solid ratios (LSR, 4 g/g and 8 g/g). The resulting hemicellulosic liquors (liquid phase) were denominated according to their potential xylose (g/L), i.e., Liquor 29X_Pot_, 32X_Pot_ and 54X_Pot_, accordingly. Solid phase was composed mainly by cellulose (measured as glucan) and lignin. As seen in Table 1, low xylan content, ranging between 5.74 and 10.80%, remained in the solid phase, which corresponded to 81–89% of xylan solubilization. After treatment, between 71.30 and 83.35% of xylan was recovered as XOS and xylose in the liquid phase. On the other hand, between 0.74 and 1.34% of xylan was degraded into furfural. XOS were the major component in the liquid phase, corresponding to 65–67% of total identified compounds. In the treatments at LSR of 8 g/g, similar concentration of XOS were obtained at the two severities evaluated (3.67 and 3.99). Nevertheless, maximal recovery of xylan as sum of XOS and xylose (32 g/L) was obtained at severity of 3.99 (Liquor 32X_Pot_). Moreover, glucooligosaccharides were also quantified achieving a concentration in the range of 1.86–3.36 g/L (Table 1). Regarding degradation compounds (such as furfural and hydroxymethylfurfural-HMF), their concentration was higher at severity of 3.99 than at *S*_0_ = 3.67. To evaluate the capacity of strains at different substrate concentrations, liquid-to-solid ratio in the hydrothermal pretreatment was reduced to 4 g/g to obtain a liquor with higher XOS and xylose concentration, achieving a xylose potential (measured as sum of XOS and xylose) of 54 g/L (Liquor 54X_Pot_). Nevertheless, undesired compounds, including acetic acid, furfural and HMF, were also increased in this hemicellulosic liquor, even with the decrease of the treatment severity to 3.79 (Table 1), which may have an inhibitory effect on yeast growth [20].

**Table 1:** Composition of the pretreated corn cob and hemicellulosic liquors resultant of different hydrothermal treatments

Preteatment conditions	*S*_0_ = 3.67 and LSR = 8 g/g	*S*_0_ = 3.99 and LSR = 8 g/g	*S*_0_ = 3.78 and LSR = 4 g/g
Solid yield (g of pretreated corn cob/100 g of corn cob)	57	42	57
Pretreated corn cob composition (g of component/100 g of pretreated corn cob)			
Glucan	49.6 ± 0.60	63.32 ± 2.20	54.46 ± 1.59
Xylan	10.05 ± 0.10	5.74 ± 0.12	10.80 ± 0.30
Arabinan	1.21 ± 0.10	0.39 ± 0.02	0.66 ± 0.02
Acetyl groups	0.53 ± 0.01	0.52 ± 0.06	0.87 ± 0.07
Klason Lignin	19.4 ± 0.60	18.53 ± 0.56	21.58 ± 0.88
Liquid-phase composition (g/L)			
Liquors	29X_Pot_	32X_Pot_	54X_Pot_
Glucose	0.435 ± 0.012	0.353 ± 0.055	0.547 ± 0.023
Xylose	2.00 ± 0.07	5.01 ± 0.02	5.49 ± 0.22
Arabinose	1.46 ± 0.04	1.33 ± 0.02	2.37 ± 0.10
Acetic acid	1.06 ± 0.04	1.59 ± 0.01	2.42 ± 0.08
Hydroxymethylfurfural (HMF)	0.0734 ± 0.0024	0.123 ± 0.000	0.166 ± 0.007
Furfural	0.408 ± 0.011	0.649 ± 0.000	1.40 ± 0.04
Glucooligosaccharides	2.04 ± 0.06	1.86 ± 0.06	3.36 ± 0.04
Xylooligosaccharides	26.5 ± 0.9	26.7 ± 0.9	48.8 ± 1.4
Arabinooligosaccharides	1.63 ± 0.19	1.25 ± 0.06	2.43 ± 0.23
Acetyl groups	2.29 ± 0.11	2.25 ± 0.14	4.6 ± 0.34

Treatments were performed under non-isothermal conditions

*S*_0_: severity; LSR: liquid-solid ratio

### Evaluation of engineered yeast strains’ hydrolytic capacity on hemicellulose derived compounds

**Fig. 1:**
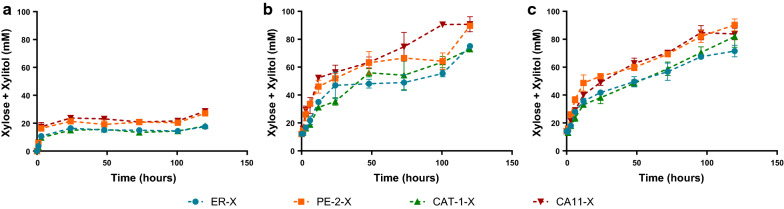
Saccharification capacity of the strains ER-X, PE-2-X, CAT-1-X and CA11-X. Assays were performed in xylan from beechwood (**a**) and in corn cob liquor 29X_Pot_ (**b**, **c**) at 40 °C. Inoculum for hydrolysis analyses was normalized by Units of xylanase activity (140 U/L; **a**, **b**) or by grams of fresh yeast (10 g/L; **c**). Data represents the average ± SD from at least two biological replicates

*Aspergillus aculeatus* β-glucosidase 1 (BGL1), *Aspergillus oryzae* β-xylosidase A (XYLA) and *Trichoderma reesei* endoxylanase II (XYN), enzymes previously used in *S. cerevisiae* for the degradation of hemicellulosic fractions [6, 8], were displayed in the cell surface of the commercial bioethanol strain Ethanol Red, of the isolates from first generation bioethanol plants in Brazil, PE-2 and CAT-1 strains, and of an isolate from “cachaça” fermentation from Brazil, CA11. While XYN and XYLA are required for the degradation of xylan and XOS, the BGL1 enzyme was expressed to saccharify the glucooligosaccharides (GOS) also present in the hemicellulosic liquors (Table 1). Then, the capacity of the different *S. cerevisiae* industrial strains as whole cell biocatalysts for the saccharification of two hemicellulose derived compounds with different polymerization degrees (commercial beechwood xylan and hemicellulosic liquor obtained from hydrothermal treatment of corncob as described above) was evaluated. The modified strains, ER-X, PE-2-X, CAT-1-X and CA11-X, were characterized in terms of xylanase activity at 30 and 40 °C (see Additional file 1: Table S1) and capacity of saccharification of xylan from beechwood and corn cob hemicellulosic liquor 29X_Pot_ (Fig. 1, Table 2).

**Table 2:** Saccharification parameters of the different *S. cerevisiae* strains displaying hemicellulolytic enzymes

Substrate	Potential xylose (mM)	Inoculum	Strain	Xylose_f_ (mM)	Xylitol_f_ (mM)	Saccharification yield (%)
Xylan from beechwood	68.1	140 U of xylanase activity/L	ER-X	7.85 ± 0.40	9.72 ± 0.26	25.8 ± 1.0
			PE-2-X	17.1 ± 0.1	9.98 ± 0.39	39.8 ± 0.4
			CAT-1-X	11.4 ± 0.2	6.60 ± 0.51	26.4 ± 1.1
			CA11-X	14.5 ± 0.1	14.1 ± 0.8	42.1 ± 1.1
Liquor 29X_Pot_ (*S*_0_ = 3.67 and LSR = 8 g/g)	184	140 U of xylanase activity/L	ER-X	58.5 ± 0.6	16.5 ± 0.5	40.8 ± 0.6
			PE-2-X	78.2 ± 0.0	11.5 ± 0.3	48.7 ± 0.2
			CAT-1-X	62.9 ± 0.2	9.94 ± 0.14	39.6 ± 0.2
			CA11-X	67.8 ± 3.2	22.9 ± 0.6	49.3 ± 2.1
		10 g of fresh yeast/L	ER-X	64.6 ± 1.6	24.4 ± 1.1	38.9 ± 1.6
			PE-2-X	96.7 ± 5.9	13.8 ± 0.9	49.1 ± 1.6
			CAT-1-X	81.1 ± 0.1	14.2 ± 0.1	44.5 ± 3.0
			CA11-X	86.4 ± 3.0	23.4 ± 0.7	45.5 ± 1.6

Saccharification assays were performed in beechwood xylan or corn cob liquor 29X_Pot_ at 40 °C. Data represents the average ± SD from two biological replicates

*S*_0_: severity; LSR: liquid-solid ratio

As expected [21], the xylanase activity of the strains was higher at 40 °C than at 30 °C, being almost twofold higher for the ER-X strain (see Additional file 1: Table S1). Also, the ER-X strain presented the higher xylanase activity (see Additional file 1: Table S1). However, when evaluating the kinetic profile of saccharification of xylan from beechwood and XOS of corn cob liquor, PE-2-X and CA11-X were the strains capable of higher xylose release (measured as sum of xylose and xylitol, expressed in mM) (Fig. 1a–c). It should be noted that xylitol is produced from xylose by the action of unspecific aldose reductases natively present on *S. cerevisiae* strains and that this production varies among yeast strains [5, 16]. Thus, for analyzing xylan degrading capacity among different strains, the concentrations of xylose together with xylitol was considered (Fig. 1b–d). As seen, all the constructed strains were able to saccharify both substrates. Nevertheless, differences were observed among biocatalysts and substrates. The maximum saccharification of corn cob liquor was obtained with PE-2-X and CA11-X, reaching 49% of the potential xylose, while ER-X reached only 39% (Table 2). The incomplete hydrolysis can be a result of the heterogeneous structure of the beechwood/corn cob-derived xylan [22] in addition to product inhibition that may affect the cell-surface displayed enzymes due to xylose accumulation [7]. The hydrolysis of xylan is also known to be dependent of the raw material of origin and extraction method, varying in molecule length, degree of branching and presence of side groups [23]. This can be clearly observed in the different saccharification profiles observed between the commercial beechwood-derived xylan and the corn cob liquor (Fig. 1), with the maximum saccharification of beechwood xylan being only 42% (Table 2). In fact, the total degradation of xylan requires the action of xylanolytic enzymes other than xylanase and xylosidade to remove side chains (such as arabinose, glucuronic acid, acetyl groups) from the xylan backbone. Nevertheless, the activity of these accessory enzymes, while increasing the amount of released xylose, would also cleave acetyl groups which would result in acetic acid accumulation in the medium with the consequent negative effects on yeast viability [20]. Different inoculum of the strains were also tested in corn cob liquor 29X_Pot_: in one experiment the strains were inoculated by xylanase activity (Fig. 1b) and in the other by wet cell weight (Fig. 1c). The similar xylan-degrading abilities observed with the same strain in different inoculums concentrations, also indicates that there is no enzyme/substrate ratio limitation.

Differences between the hydrolytic performance of the different strains (Fig. 1) were expected, as the efficiency of cell-surface display is dependent of several host-related factors [24], which have already been reported to vary among *S. cerevisiae* strains: e.g., secretory capacity [18], cell wall composition [25, 26], expression levels of host-cell genes [27]. Furthermore, yeast cell size is another source of variability, as larger cells have a lower total superficial cell area available for enzyme display per unit biomass. It should also be noted that the strains presenting higher hemicellulolytic capacity (CA11-X and PE-2-X, Fig. 1) were not the ones presenting higher XYLA and XYN activity values (see Additional file 1: Table S1), supporting the necessity of making the selection of appropriate yeast strain backgrounds in process-like conditions. Another factor to be taken into consideration is the flocculation ability, a process that is mediated by lectin-like receptors present in the cell surface which bind mannose residues in adjacent cells creating clusters of thousands of cells [28], but have also been reported to bind to a wide range of sugars [25]. Accordingly, the CA11 strain, being a flocculant strain, will present an high number of lectin-like receptors in the cell surface [25], that may potentiate the binding of the yeast to sugar residues in xylan/XOS, and benefit the hydrolysis in the long term due to substrate proximity. Furthermore, different predisposition to convert xylose into xylitol, normally observed between *S. cerevisiae* strains [16], may alleviate product inhibition of xylanase and xylosidase by xylose, increasing the overall degradation of xylan.

### Evaluation of engineered yeast strains’ capacity for direct production of ethanol from hemicellulosic liquor

To further evaluate the potential of the different yeast strains to directly produce ethanol from hemicellulose, i.e., degrade xylan and ferment the resulting xylose, they must be modified to be able to consume xylose. Recently, the oxidoreductase (xylose reductase-XR/xylitol dehydrogenase-XDH [29]) and the isomerase (xylose isomerase-XI, with a low susceptibility to xylitol inhibition [30]) xylose-consumption pathways were simultaneously expressed in robust industrial *S. cerevisiae* strains, improving the ethanol yield from a non-detoxified corn cob hydrolysate, with coupled higher furan detoxification and lower xylitol production, in comparison with the single expression of XR/XDH or XI [31]. Thus, in this work we opt for the simultaneous expression of the oxidoreductase and the isomerase metabolic pathways. Furthermore, to favour both saccharification and fermentation, the strains should be metabolic active at the optimal conditions previously described for the cell-surface displayed XYN, namely pH 5 and temperature of 40 °C [21]. In fact, the ability to ferment at higher temperatures has already been identified as a crucial trait for CBP yeast [1], and in this sense, the use of thermotolerant industrial *S. cerevisiae* strains is an advantage. Considering the trait variability among industrial strains, and the recognized necessity of a tailor-made design of yeast (considering process conditions and specific raw material) [16, 32], the strains ER-X, PE-2-X, CAT-1-X and CA11-X were evaluated in terms of capacity to ferment an hydrothermally pretreated corn cob liquor (supplemented with synthetic glucose) at 40 °C (see Additional file 1: Fig. S1). All the strains were capable of fermenting in these conditions; however, the PE-2-X strain produced lower ethanol titers (see Additional file 1: Fig. S1), being incapable of consuming all the glucose present in the medium (data not shown). Considering this poor performance, only the ER-X, CAT-X and CA11-X strains were further modified with the oxidoreductase and isomerase pathways for xylose consumption, resulting in strains ER-X-2P, CAT-X-2P and CA11-X-2P. Also important to note is the fact that the growth ability of the strains was not affected by the cell-surface display of the hemicellulases (see Additional file 1: Fig. S2) and that the integrations at the δ-sequences were found to be stable (see Additional file 1: Fig. S3).

**Fig. 2:**
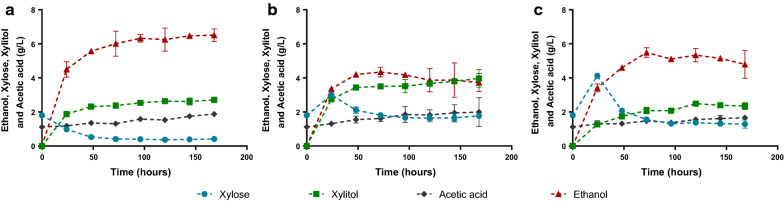
Consolidated bioprocessing profiles of the strains ER-X-2P (**a**), CAT-1-X-2P (**b**) and CA11-X-2P (**c**). Assays were performed in corn cob liquor 29X_Pot_ with an inoculum of 50 g/L fresh yeast. Data represents the average ± SD from two biological replicates

The ER-X-2P, CAT-X-2P and CA11-X-2P strains were capable of producing ethanol directly from the corn cob liquor 29X_Pot_ at 40 °C (Fig. 2, Table 3), with ER-X-2P reaching the highest ethanol titer of 6.51 g/L (corresponding to an ethanol yield of 0.247 g/g of potential sugar), consuming almost all the xylose that was liberated in the medium (Fig. 2a, Table 3). On the other hand, the CAT-X-2P and CA11-X-2P strains produced significantly lower levels of ethanol (Fig. 2b, c, Table 3) with an accumulation of xylose at 24 h of fermentation. This indicates that despite the xylan-degrading capacity of these strains, their aptitude to uptake and ferment the liberated xylose is inferior in comparison with ER-X-2P.

**Table 3:** CBP and SSF parameters of the different xylose-consuming *S. cerevisiae* strains in corn cob liquors

Corn cob liquor	Potential xylose (g/L)	Strain (inoculum)	Glucose_i_ (g/L)	Xylose_i_ (g/L)	Xylose_f_ (g/L)	Ethanol_f_ (g/L)	Xylitol_f_ (g/L)	Acetic acid_i_ (g/L)	Acetic acid_f_ (g/L)	*Y*_xyol/potential xyl_ (g/g)	*Y*_et/potential sugar_ (g/g)
Liquor 29X_Pot_ (*S*_0_ = 3.67 and LSR = 8 g/g)	24.2	ER-X-2P (50 g/L)	0.392 ± 0.003	1.80 ± 0.02	0.418 ± 0.016	6.51 ± 0.26	2.71 ± 0.00	1.13 ± 0.02	1.88 ± 0.02	0.112 ± 0.000	0.247 ± 0.010
		CAT-1-X-2P (50 g/L)	0.392 ± 0.003	1.80 ± 0.02	1.77 ± 0.01	3.73 ± 0.37	3.97 ± 0.37	1.13 ± 0.02	1.99 ± 0.60	0.164 ± 0.015	0.142 ± 0.014
		CA11-X-2P (50 g/L)	0.392 ± 0.003	1.80 ± 0.02	1.29 ± 0.17	4.80 ± 0.58	2.35 ± 0.14	1.13 ± 0.02	1.66 ± 0.09	0.0971 ± 0.0059	0.182 ± 0.022
Liquor 32X_Pot_ (*S*_0_ = 3.99 and LSR = 8 g/g)	31.6	ER-X-2P (50 g/L)	0.293 ± 0.003	5.02 ± 0.11	2.11 ± 0.28	8.15 ± 0.54	4.59 ± 0.42	1.81 ± 0.00	2.17 ± 0.20	0.145 ± 0.013	0.240 ± 0.016
		ER-X-2P (100 g/L)	0.293 ± 0.003	5.02 ± 0.11	0.873 ± 0.019	11.1 ± 0.2	4.75 ± 0.13	1.81 ± 0.00	2.37 ± 0.01	0.150 ± 0.004	0.328 ± 0.005
Liquor 54X_Pot_ (*S*_0_ = 3.78 and LSR = 8 g/g)	50.7	ER-X-2P (100 g/L)	0.487 ± 0.010	4.62 ± 0.02	14.0 ± 0.8	4.23 ± 0.04	6.19 ± 0.67	2.53 ± 0.01	3.91 ± 0.34	0.122 ± 0.013	0.0778 ± 0.0008
Whole slurry (liquor 54X_Pot_ with 5% solid loading)	50.7 + 6.13	ER-X-2P (100 g/L)	0.487 ± 0.010	4.62 ± 0.02	14.4 ± 1.0	4.20 ± 0.15	6.41 ± 0.21	2.53 ± 0.01	3.72 ± 0.32	0.113 ± 0.004	0.0463 ± 0.0017
Liquor 32X_Pot_ (*S*_0_ = 3.99 and LSR = 8 g/g) plus 0.017% (v/v) hemicellulase	31.6	ER-2P (100 g/L)	0.466 ± 0.151	5.15 ± 0.19	0.976 ± 0.095	4.65 ± 0.16	0.760 ± 0.022	1.90 ± 0.16	3.38 ± 0.08	0.0240 ± 0.0007	0.137 ± 0.005
		ER-X-2P (100 g/L)	0.466 ± 0.151	5.15 ± 0.19	6.55 ± 0.25	6.26 ± 0.06	5.40 ± 0.08	1.90 ± 0.16	3.41 ± 0.19	0.171 ± 0.003	0.184 ± 0.002
Liquor 32X_Pot_ (*S*_0_ = 3.99 and LSR = 8 g/g) plus 0.050% (v/v) hemicellulase		ER-2P (100 g/L)	0.466 ± 0.151	5.15 ± 0.19	1.02 ± 0.00	6.26 ± 0.10	0.854 ± 0.125	1.90 ± 0.16	3.65 ± 0.22	0.0270 ± 0.0040	0.184 ± 0.003
		ER-X-2P (100 g/L)	0.466 ± 0.151	5.15 ± 0.19	8.65 ± 0.02	6.00 ± 0.16	5.01 ± 0.08	1.90 ± 0.16	3.60 ± 0.24	0.159 ± 0.003	0.176 ± 0.005

Assays were performed at 40° C and oxygen-deprived conditions. Data represents the average ± SD from two biological replicates

*S*_0_: severity; LSR: liquid-to-solid ratio; *Y*_et/potential sugar_: ethanol yield from potential sugars; *Y*_xyol/potential xyl_: xylitol yield from potential xylose

This superior performance of ER-X-2P is in accordance with previous reports of Ethanol Red excellent fermentation capacity, robustness, stress [33] and temperature tolerance [34]. In fact, Ethanol Red has recently been successively used as host for consolidated bioprocessing (CBP) for first generation bioethanol using raw starch [1], with the present work showing this strain applicability also for a more challenging CBP for second generation hemicellulosic ethanol.

### Consolidated bioprocessing of corn cob hemicellulosic liquor: evaluation of pretreatment conditions and inoculum size

**Fig. 3:**
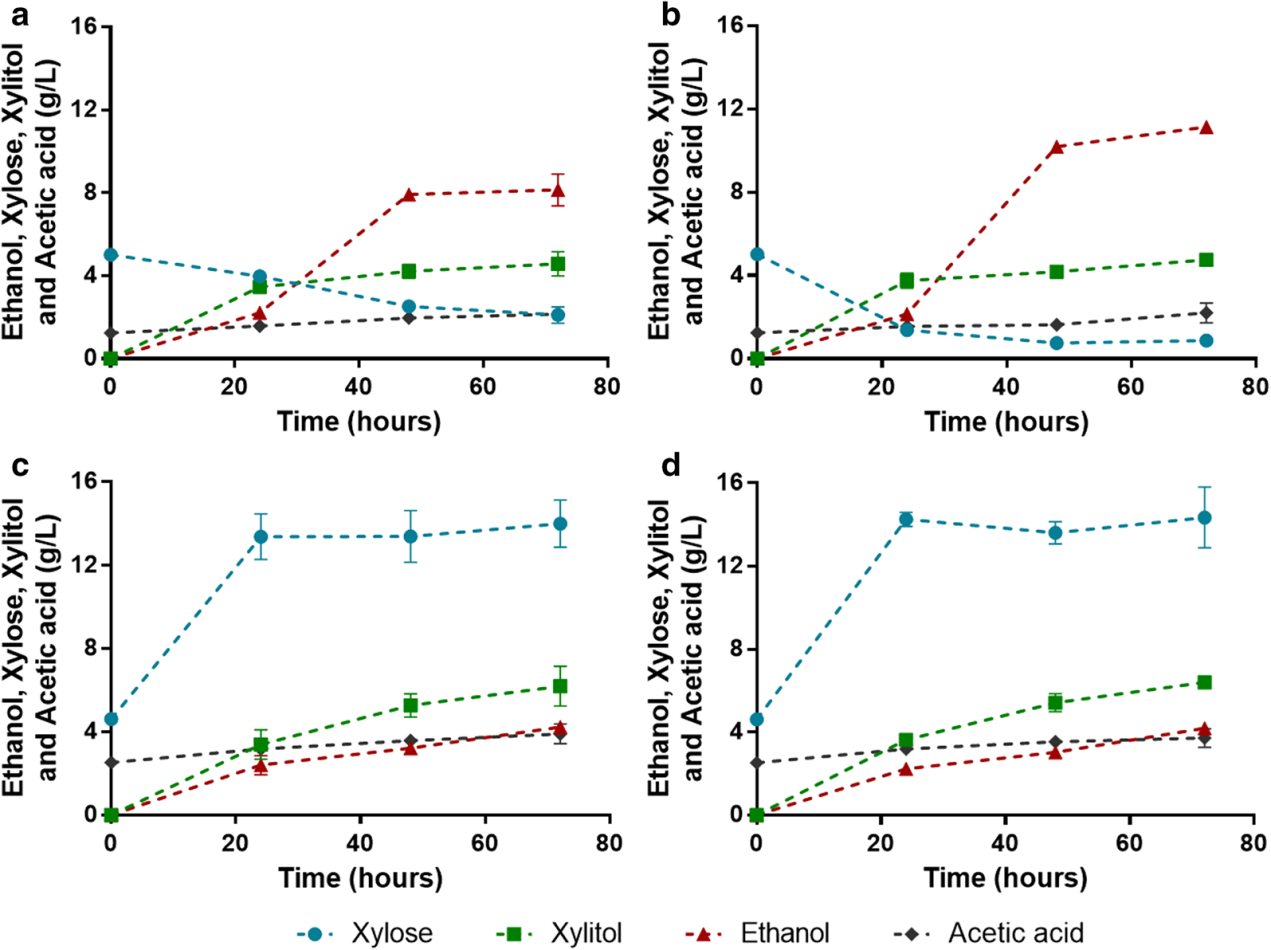
Consolidated bioprocessing profiles of the strain ER-X-2P. Assays were performed in corn cob liquor 32X_Pot_ (**a**, **b**), corn cob liquor 54X_Pot_ (**c**) and corn cob liquor 54X_Pot_ with 5% solids (**d**) with an inoculum of 50 g/L fresh yeast (**a**) or 100 g/L fresh yeast (**b**–**d**). Data represents the average ± SD from two biological replicates

As observed in Table 1, the increase of severity of treatment implies a higher solubilization of xylan in the liquid phase as XOS and xylose [35] which implies higher potential sugars to be fermented into ethanol. Therefore, the corn cob liquor obtained at severity of 3.99 (Liquor 32X_Pot_) was used to evaluate the role of inoculum size of ER-X-2P for simultaneous saccharification of XOS and fermentation into ethanol (Fig. 3a, b). In fact, this increase in pretreatment severity (3.99) allowed a higher ethanol production of 8.15 g/L, when compared with the 6.51 g/L obtained with the same strain and inoculum in liquor 29X_Pot_ (Figs. 2a and 3a, same strain and inoculum quantity). Furthermore, it was observed that increasing the inoculum from 50 to 100 g wet cells/L (7.5 to 15 g dry cell weight DCW/L), an inoculum concentration in the order of magnitude of the normally reported for CBP strains [8, 36], improved the overall process (Fig. 3a, b) and increased the ethanol titer from 8.15 to 11.1 g/L (corresponding to an increase of 36% in ethanol concentration) and the ethanol yield from 0.240 to 0.328 g per g of potential sugar (Table 3). These results represent the highest ethanol concentration obtained so far directly from an hemicellulosic liquor through a CBP microorganism. In fact, the ethanol titer attained with this CBP approach is superior to the ones previously reported for fermentation of non-detoxified corn cob hydrolysates obtained by acid hydrolysis [16, 31, 37]. Furthermore, the ethanol yield of 0.328 g/g is comparable to results previously obtained from simultaneous saccharification and co-fermentation (SSCF) of a whole slurry (containing cellulose and hemicellulose and using commercial cellulases and hemicellulases) with an Ethanol Red strain modified for xylose consumption (yields of 0.32 and 0.28 g/g at 32 and 39 °C, respectively) [33]. This highlights the robustness of the strain constructed in this study as even with the additional modifications for the display of enzymes on the cell surface, it was capable of maintaining ethanol yields (in a fermentation media mainly composed of xylose, where normally, the obtained yields are lower than in the presence of glucose) similar to those of a less modified strain in a media mainly containing both xylose and glucose.

Another strategy tested to increase ethanol concentration was to increase the potential of fermentable xylose derived from the corn cob pretreatment by reducing the liquid-to-solid ratio to 4 g/g, allowing a recovery of 48.8 g/L of XOS (Liquor 54X_Pot_, Table 1). However, the CBP of this liquor with ER-X-2P (Fig. 3c) resulted in lower ethanol titers than the ones previously obtained (even when using lower inoculums, Figs. 2a, 3a, b). This poor fermentative performance is explained by a significant increase in the inhibitory composition of the liquor resulting from the decrease in LSR, with liquor 54X_Pot_ presenting more than twofold the concentrations of acetic acid, HMF and furfural when comparing to liquor 29X_Pot_ (Table 1). Considering these, the pretreatment conditions for this process must be carefully defined to maximize xylan extraction while maintaining inhibitors concentration at a non-toxic level for the yeast strain.

Additionally, CBP with ER-X-2P was also performed by mixing corn cob liquor 54X_Pot_ with corn cob pretreated solids (obtaining a whole slurry as substrate), which contain 10.8 g of xylan per 100 g of pretreated corn cob (Table 1, Fig. 3d). Similar saccharification and fermentation profiles were observed for both experiments (with and without solids addition (Fig. 3c, d). This behavior shows that this strain was incapable of degrading the xylan remaining in the pretreated corn cob solid (Table 1), probably due to a lack of accessibility which would be solved in the case of a simultaneous degradation of cellulose (by addition of cellulases or of a cellulase-degrading strain). This assay also shows that there is no unproductive binding of the cell-surface-displayed enzymes to the lignin present in the solid fraction, a common problem in hydrolysis of lignocellulosic biomass [38], since equal saccharification yields from liquid phase (containing XOS) were obtained. With these results this strain is shown to be suitable for applications in integrated lignocellulosic processes (e.g., co-culture with cellulolytic strains for whole slurry CBP). Furthermore, this cell-surface display approach to CBP also facilitates the recycling of hydrolytic enzymes, an important technology for the development of economically viable processes [39]. In fact, cell recycling was attempted in CBP with ER-X-2P, and despite the gradual loss of yeast cell fermentative capacity, the hydrolytic activity of the cell-surface displayed enzymes was maintained after 2 cycles of recycling, releasing similar concentrations of xylose (see Additional file 1: Fig. S4). This also shows the advantages of the cell-surface display approach to express enzymes, and the suitability of these modified strains to function as whole-cell biocatalysts even with the loss of its native metabolic activity.

### Evaluation of commercial hemicellulase addition

**Fig. 4:**
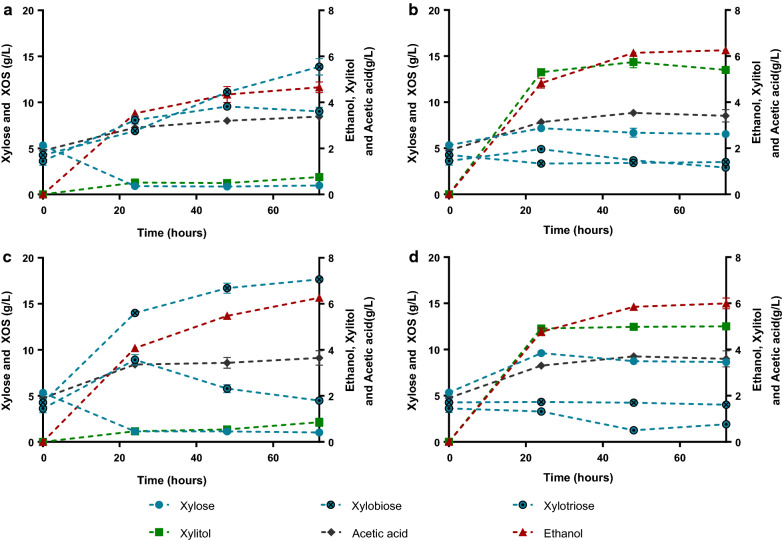
Simultaneous saccharification and fermentation profiles of the strains ER-2P (**a**, **c**) and ER-X-2P (**b**, **d**). Assays were performed in corn cob liquor 32X_Pot_ with addition of commercial hemicellulase (0.017% (v/v): **a**, **b**; 0.050% (v/v): **c**, **d**) with an inoculum of 100 g/L fresh yeast. Data represents the average ± SD from two biological replicates

**Fig. 5:**
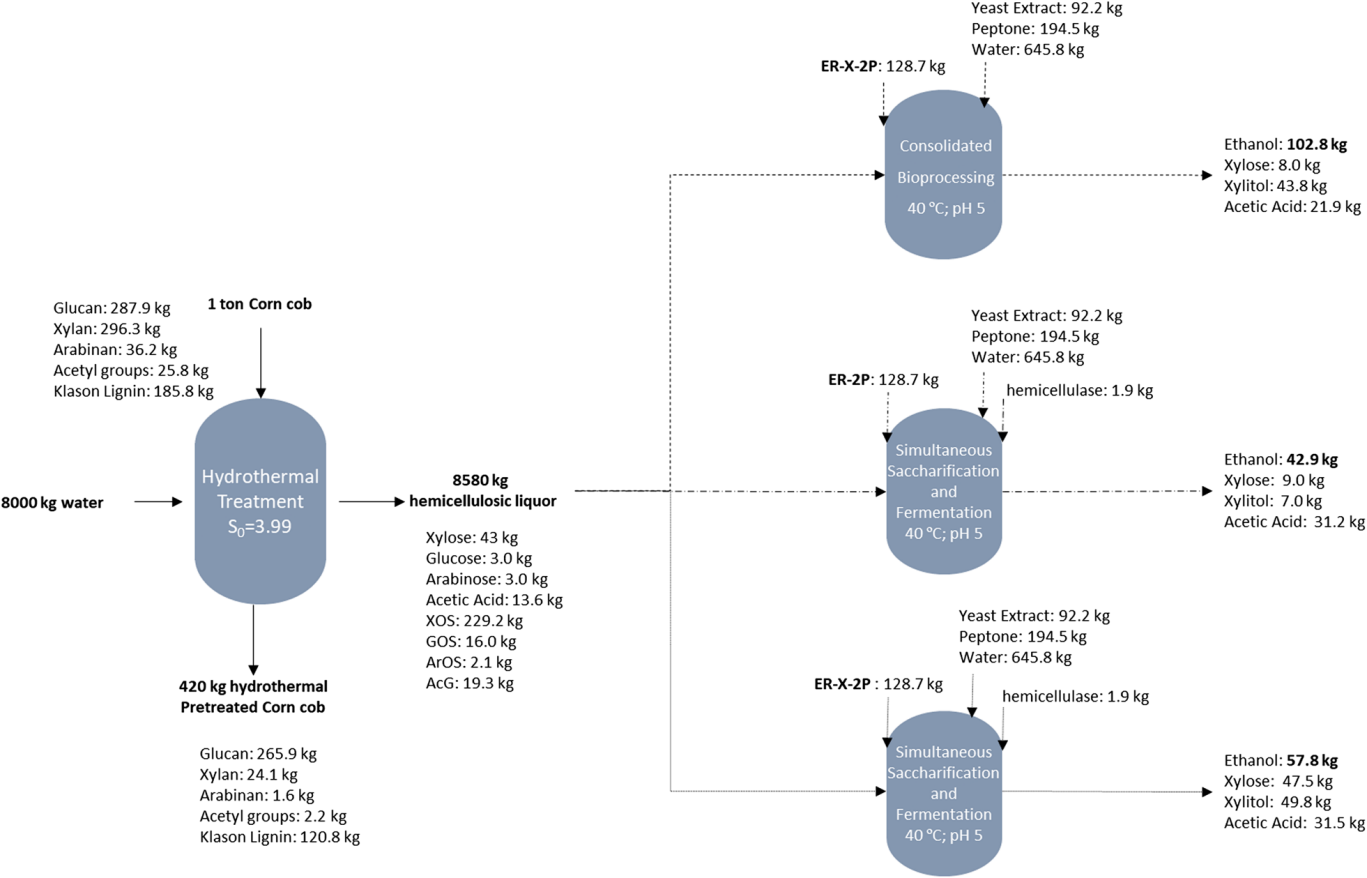
Mass balance of consolidated bioprocessing and simultaneous saccharification and fermentation of the hemicellulosic liquor 32X_Pot_

The SSF of corn cob liquor 32X_Pot_ was also performed with the ER-2P strain (modified for xylose consumption with the XR/XDH and XI pathways but without the cell-surface display of enzymes) and with the addition of reduced quantities of the commercial cocktail of hemicellulases Cellic HTec2 (Fig. 4a, c), to compare with the direct production of ethanol from hemicellulose with only the ER-X-2P strain (Fig. 3b). Two concentrations of commercial hemicellulases were tested: 0.050% (v/v), to equal the xylanase activity normally added with the inoculum of ER-X-2P; and 0.017% (v/v), to evaluate the effect of a two-third reduction of enzyme quantities (done to reduce process costs). Nevertheless, even with the higher concentration of commercial hemicellulase, the ethanol titers obtained with this method were lower than with the CBP process, 4.65 and 6.26 g/L compared to the previously attained 11.1 g/L (Table 3). Considering the high levels of xylobiose and xylotriose accumulated and the low levels of xylose throughout the experiments (Fig. 4a, c), it is clear that the commercial cocktail, while being capable of producing XOS with low degree of polymerization, is incapable to release xylose at the rate that it is being consumed by ER-2P. This supports the already reported deficit of xylosidase activity in commercial hemicellulase cocktails [8, 40], and an efficient hydrolysis of this material would require the use of a tailored mixture (e.g., by addition of more xylosidase) or optimized more recent cocktail formulations. On the other hand, when performing the SSF with addition of commercial cocktail but with the ER-X-2P strain (Fig. 4b, d), the concentration of xylobiose and xylotriose was maintained low throughout the experiment, showing that the xylanolytic strain compensates the low xylosidase activity of the commercial hemicellulases. Furthermore, the important role of ER-X-2P for the degradation of hemicellulose is also shown, as it produced similar ethanol titers in SSF with both concentrations of commercial hemicellulases (Table 3, Fig. 4b, d), while with ER-2P the ethanol titer was significantly lower when less hemicellulase cocktail was added (Table 3, Fig. 4a, c). Nevertheless, all the SSF experiments of corn cob liquor 32X_Pot_ with addition of commercial hemicellulases resulted in low ethanol titers (≤ 6.26 g/L, Fig. 4), while the CBP of the same liquor with only ER-X-2P produced at least 1.77-fold higher concentrations (Fig. 3b, Table 3). This low performance, may be attributed to the levels of acetic acid that were released throughout the experiment (mainly in the first 24 h, Fig. 4), due to the fact that, in contrary to the ER-X-2P strain, the commercial cocktail contains other hemicellulases capable of removing xylan side chains, such as acetyl xylan esterase (EC 3.1.1.72). In fact, while in the CBP of liquor 32X_Pot_ with no enzyme addition, the increase of this weak acid was minimal (1.3-fold, Fig. 3b), with commercial enzymes supplementation the acetic acid concentrations increased more than 1.8-fold in the 72 h of SSF (Fig. 4), which clearly had a negative impact in the yeast cell fermentative capacity. Summing up, the use of this commercial cocktail in conjugation with ER-X-2P strain, while increasing the amount of released xylose, also significantly increased the acetic acid concentration of the medium, reducing the overall ethanol production in comparison with CBP using only the yeast strain ER-X-2P (Fig. 5). In fact, an overall balance of corn cob processing for hemicellulosic ethanol production considering the results obtained in this work with Liquor 32X_Pot_ (Fig. 5) clearly shows the advantage of CBP: while the SSF process may produce a maximum of 57.8 kg of ethanol from 1 ton of corn cob requiring 1.9 kg of commercial enzymatic cocktail, the sole use of ER-X-2P yeast strain as biocatalyst allows the production of 102.8 kg of ethanol from the same amount of raw material, without addition of exogenous enzymes. Additionally, the SSF with the ER-2P strain allows the attainment of only 42.9 kg of ethanol/ton of corn cob, also highlighting the importance of using the hemicellulolytic strains. These results show that the use of engineered industrial *Saccharomyces cerevisiae* strains as whole-cell biocatalysts for the saccharification of corn cob-derived hemicellulosic liquor is advantageous for ethanol production in comparison with the use of commercial enzymatic cocktails. Finally, it should be noted that higher ethanol titers need to be obtained to decrease the distillation costs and make CBP an economical feasible process. For that both cellulosic and hemicellulosic fractions of lignocellulose must be used to increase the load of fermentable carbon, and in this sense, the improvement of the ethanol titers from the hemicellulosic fraction represents a step forward towards the attainment of efficient CBP processes.

## Conclusions

These results show the highest ethanol concentration reported from direct conversion of hemicellulosic liquors by *S. cerevisiae*, which are also higher than ethanol values obtained from corn cob-derived hemicellulose after acid post hydrolysis. In this sense, the use of engineered industrial *Saccharomyces cerevisiae* strains as whole-cell biocatalysts is shown to be a suitable alternative to commercial enzymatic cocktails or chemical hydrolysis and represents a greener and economical approach to produce hemicellulosic ethanol from corn cob biomass. Additionally, the potential of robust industrial *S. cerevisiae* strains as hosts for the design of whole-cell biocatalysts is demonstrated, paving the way for the construction of more efficient strains for consolidated bioprocessing in lignocellulosic biorefineries.

## Methods

### Yeast strains and plasmid construction

*Saccharomyces cerevisiae* strains and main plasmids used in this work are listed in Table 4 and primers and plasmids used for cloning steps are presented on Additional file 1: Table S2. *Escherichia coli* DH5α was used for plasmid construction, propagation and maintenance. The integrative plasmid pI23-BGL1-kanMX for expression of *Aspergillus aculeatus* β-glucosidase 1 (BGL1; EC 3.2.1.21; Accession P48825.1) was obtained from the plasmid pIBG-SSA by substitution of the *HIS3* for the integration region 23 (3′ non-coding region of genes *YCL054W* and *YCL052C*, chromosome III) [41] and the *kanMX* resistance marker. The plasmid pI5-XylA-NatX for expression of *Aspergillus oryzae* β-xylosidase A (XYLA; EC 3.2.1.37; Accession OOO12801.1) was obtained from the plasmid pIK-BX-SSS by substitution of the *LYS2* for the integration region I5 (3′ non-coding region of genes *YLL055W* and *YLL054C*, chromosome XII) [3] and the *natMX* resistance marker. The δ-integration plasmid pδW-XYN-kanMX for expression of *Trichoderma reesei* endoxylanase II (XYN; EC 3.2.1.8; Accession XP_006968947.1) was constructed from the pδW-EX-SSS by substitution of the *TRP1* marker for the kanMX-UkG1 cassette, containing both the *kanMX* resistance marker and the green fluorescent protein mUkG1 [42] for monitoring copy number integration. Plasmid assembling steps were performed with the In-Fusion HD Cloning Kit (Clontech, Mountain View, CA, USA). The gene cassettes used for the cell-surface expression of hemicellulases were previously optimized [43, 44] and consist of: coding sequences of *SED1* promoter, *SED1* secretion signal, and *SAG1* anchoring domain for BGL1 expression; and coding sequences of *SED1* promoter, *SED1* secretion signal, and *SED1* anchoring domain for expression of XYLA and XYNII. The plasmids pI23-BGL1-kanMX, pI5-XylA-NatMX and pδW-XYN-kanMX were linearized with BstZ17I, SpeI and AscI, respectively, and were sequentially transformed by lithium acetate [45] and integrated in the different yeast strains. The transformants were selected on YPD plates (10 g/L yeast extract, 20 g/L peptone, 20 g/L glucose and 20 g/L agar) containing 300 μg/mL of G418 (pI23-BGL1-kanMX and pδW-XYN-kanMX) or 100 μg/mL of clonNAT (pI5-XylA-NatMX) and were preserved at 4 °C on YPD plates. Before integration of pδW-XYN-kanMX, the *kanMX* marker inserted with the pI23-BGL1-kanMX integration was removed using the CRE-loxp recombinase system [46]. After each integration, transformants of each of the different yeast hosts were screened, and the transformant presenting higher enzymatic activity was selected for further work (data not shown), and were denominated ER-X, PE-2-X, CAT-1-X and CA11-X. For xylose consumption the plasmid pMEC1049 + XI, for expression of both the oxidoreductase and the isomerase pathways [31], was introduced by lithium acetate transformation [45] on the yeast strains displaying the hemicellulolytic enzymes, originating the strains ER-X-2P, CAT-1-X-2P and CA11-X-2P, and also on the ER wild type strain, originating the strain ER-2P. The transformants were selected on YPD plates containing 300 μg/mL of hygromycin and were preserved at 4 °C on YPX plates (10 g/L yeast extract, 20 g/L peptone, 20 g/L xylose and 20 g/L agar) containing 300 μg/mL of hygromycin to maintain selective pressure.

**Table 4:** *Saccharomyces cerevisiae* strains and main plasmids used in this study

	Relevant features	Source
*Saccharomyces cerevisiae*		
Ethanol Red (ER)	Commercial ethanol yeast	Fermentis, S. I. Lesaffre, Lille
PE-2	Isolated from a Brazilian first generation bioethanol plant	[14, 49]
CAT-1	Isolated from a Brazilian first generation bioethanol plant	[14, 49]
CA11	Isolated from a Brazilian “cachaça” fermentation processes	[13, 50]
ER-X	ER, pI23-BGL1-kanMX, pI5-XylA-NatMX, pδW-XYN-kanMX	This work
PE-2-X	PE-2, pI23-BGL1-kanMX, pI5-XylA-NatMX, pδW-XYN-kanMX	This work
CAT-1-X	CAT-1, pI23-BGL1-kanMX, pI5-XylA-NatMX, pδW-XYN-kanMX	This work
CA11-X	CA11, pI23-BGL1-kanMX, pI5-XylA-NatMX, pδW-XYN-kanMX	This work
ER-X-2P	ER-X, pMEC1049 + XI	This work
CAT-1-X-2P	CAT-1-X, pMEC1049 + XI	This work
CA11-X-2P	CA11-X, pMEC1049 + XI	This work
ER-2P	ER, pMEC1049 + XI	This work
Plasmids		
pI23-BGL1-kanMX	SED1p–SED1ss–*A. aculeatus* BGL1–SAG1a–SAG1t, *KanMX* marker, I23 integration site	This work
pI5-XylA-NatMX	SED1p–SED1ss–*A. oryzae* XYLA–SED1a–SAG1t, *NatMX* marker, I5 integration site	This work
pCRE-hyg6	CRE recombinase, *hyg6*	This work
pδW-XYN-kanMX	SED1p–SED1ss–*T. reesei* XYNII–SED1a–SAG1t, *KanMX* marker, δ sequences integration site	This work
pMEC1049 + XI	pYPK4-TEF1tp-XR (N272D)-TDH3tp-XYL2-PGI1tp-XKS1-FBA1tp-TAL1-PDC1tp, *xylA* gene from *C. phytofermentans* (HXT7p and CYCt), HphMX4	[31]

### Enzymatic activities

For determination of enzymatic activities the yeast cells were grown in YPD medium (10 g/L yeast extract, 20 g/L peptone and 20 g/L glucose) for 48 h at 30 °C (see Additional file 1: Table S1) or YPX medium (10 g/L yeast extract, 20 g/L peptone and 20 g/L xylose) for 48 h at 35 °C for xylanase activity determinations of the inoculums for SSF experiments (data not shown); the cells were collected by centrifugation at 1000 g for 5 min. β-glucosidase 1 and β-xylosidase A activities were measured at 30 °C with nitrophenyl-β-d-glucopyranoside and p-nitrophenyl-β-d-xylopyranoside (Nacalai Tesque, Inc., Kyoto, Japan), respectively, as previously described [47]. For the measurement of xylanase activity, the cells (20 g wet cells/L) were incubated in 10 g/L of xylan from beechwood (Sigma, ≥ 90% purity) in 50 mM sodium acetate buffer (pH 5.0) for 10 min at 250 rpm orbital agitation at 30 and 40 °C. The amount of reducing sugar released from the substrate was measured by the DNS method [48]. One unit of xylanase activity was defined as the amount of enzyme required to release 1 μmol of reducing sugar per minute. Dry cell weight (DCW) of the yeast strains was calculated to be 0.15-fold (ER, PE-2 and CAT-1) and 0.18-fold (CA11) that of the wet cell weight.

### Preparation of corn cob liquor

Corn cob was collected, milled and submitted to hydrothermal treatment under non-isothermal conditions, selected based on previous works [5, 19], in a 2 L stainless steel reactor (Parr Instruments Company) equipped with Parr PDI temperature controller (model 4848) at liquid-to-solid ratio of 8 g distilled water/1 g of oven dry corn cob or of 4 g distilled water/1 g of oven dry corn cob (Table 3). T_max_ of 205 °C, 211 °C and 207 °C corresponding to a severity (*S*_0_) of 3.67, 3.99 and 3.78, were used to obtain the hemicellulosic liquors, that were denominated according to their potential in xylose (g/L), i.e., Liquor29X_Pot_, 32X_Pot_ and 54X_Pot_, respectively. After treatment, the resulting solid and liquid (liquor) phases were separated by filtration. Solid phase was recovered and washed for solid yield determination. Severity (*S*_0_) was calculated by the following equation:

1 $$\begin{aligned} S_{0} = \log R_{0} = \log \left( {R_{{0_{\text{HEATING}} }} + R_{{0_{\text{COOLING}} }} } \right) \hfill \\ = { \log }\left[ {\mathop \smallint \limits_{0}^{{t_{\text{MAX }} }} { \exp }\left( {\frac{{T\left( t \right) - T_{\text{REF}} }}{\omega }} \right) \cdot {\text{d}}t} \right] + \left[ {\mathop \smallint \limits_{{t_{\text{MAX}} }}^{{t_{\text{F}} }} \exp \left( {\frac{{T{\prime }\left( t \right) - T_{\text{REF}} }}{\omega }} \right) \cdot {\text{d}}t} \right], \hfill \\ \end{aligned}$$

where *R*_0_ is the severity factor, *t*_MAX_ (min) is the time needed to achieve the target temperature *T*_MAX_ (°C), *t*_F_ (min) is the time needed for the whole heating–cooling period, and *T*(*t*) and *T*ʹ(*t*) represent the temperature profiles in the heating and cooling stages, respectively. Calculations were made using the values reported usually for *ω* and *T*_REF_ (14.75 °C and 100 °C, respectively).

### Saccharification of xylan and xylooligosaccharides from corncob liquors

Yeast cells used for saccharification were cultivated at 30 °C for 48 h, with orbital agitation (200 rpm), in YPD medium. Cells were washed with water and the saccharification assays were inoculated with 10 g of wet yeast/L or with the biomass corresponding to 140 Units of xylanase activity/L. Saccharification was carried out in 50 mL Erlenmeyer flasks, with cotton stopper and working volume of 15 mL, in 10 g/L of xylan from beechwood in 50 mM sodium acetate buffer (pH 5.0) or in corn cob liquor 29X_Pot_ (pH adjusted to 5.0). Assays were performed at 40 °C in an orbital shaker at 150 rpm and samples were collected throughout the experiment for high-performance liquid chromatography (HPLC) analysis.

### Consolidated bioprocessing (CBP) and simultaneous saccharification and fermentation (SSF) assays

Yeast cells used for CBP/SSF were cultivated at 35 °C for 48 h, with orbital agitation (200 rpm), in YPX medium. Cells were washed with NaCl (0.9%) and the CBP/SSF assays were inoculated with 50 or 100 g of wet yeast/L (7.5 to 15 g DCW/L). CBP/SSF was carried out in 100 mL Erlenmeyer flasks, with glycerol lock to create oxygen-deprived conditions and working volume of 30 mL. Media consisted of corn cob liquor 29X_Pot_, 32X_Pot_ or 54X_Pot_; or corn cob liquor 54X_Pot_ with 5% (DCW/v) of pretreated corn cob solid fraction. All media were supplemented with YP (10 g/L yeast extract, 20 g/L peptone) and adjusted to pH 5.0. SSF of corn cob liquor 32X_Pot_ was performed with addition of 0.017% (v/v) or 0.050% (v/v) of the commercial hemicellulase Cellic HTec2 (Novozymes). Assays were performed at 40 °C in an orbital shaker at 150 rpm and samples were collected throughout the experiment for HPLC analysis.

### Analytical methods

The chemical composition of raw material and solid phases obtained after pretreatment were determined following standard methods described by NREL protocols (NREL/TP-510-42618-42622-4218). The liquid phase was analyzed directly and after posthydrolysis (4% H_2_SO_4_ at 121 °C for 20 min) allowing the quantification of monosaccharides, oligosaccharides (measured as monomers equivalents), acetic acid and furan derived compounds (furfural and HMF). Samples from corn cob treatment and from saccharification and fermentation assays were analyzed for quantification of glucose, xylose, xylitol, xylobiose, xylotriose, acetic acid, ethanol, HMF and furfural by HPLC using a Bio-Rad Aminex HPX-87H column, operating at 60 °C, with 0.005 M H_2_SO_4_ and at a flow rate of 0.6 mL/min. HMF and furfural were detected using an UV detector set at 270 nm, whereas the other compounds were detected using a Knauer-IR refractive index detector.

### Determination of fermentation parameters

Saccharification yield (%) was calculated using the following equation:

2 $${\text{Saccharification}}\;{\text{yield}} \left( \% \right) = \frac{{\left[ {{\text{Xylose}}_{\text{f}} } \right] + \left[ {{\text{Xylitol}}_{\text{f}} } \right]}}{{\left[ {{\text{Xylose}}_{\text{Pot}} } \right]}} \times 100,$$

where [Xylose_f_] is xylose concentration (mM) at the end of saccharification assay, [Xylitol_f_] is xylitol concentration (mM) at the end of saccharification assay and [Xylose_Pot_] is the potential xylose (mM) present in the media used.

Ethanol yield from potential sugars (*Y*_et/potential sugar_) was calculated using the following equation:

3 $$Y_{{{\text{et}}/{\text{potential}}\;{\text{sugar}}}} = \frac{{\left[ {{\text{Ethanol}}_{\text{f}} } \right]}}{{[{\text{Xylose}}_{\text{Pot}} \left] { + [{\text{Glucose}}_{\text{Pot}} } \right]}},$$

where [Ethanol_f_] is ethanol concentration (g/L) at the end of fermentation assay, [Xylose_Pot_] and [Glucose_Pot_] are the potential xylose (g/L) and glucose (g/L), respectively, present in the media used.

Xylitol yield from potential xylose (*Y*_xyol/potential xyl_) was calculated using the following equation:

4 $$Y_{{{\text{xyol}}/{\text{potential}}\;{\text{xyl}}}} = \frac{{\left[ {{\text{Xylitol}}_{\text{f}} } \right]}}{{[{\text{Xylose}}_{\text{Pot}} ]}},$$

where [Xylitol_f_] is xylitol concentration (g/L) at the end of fermentation assay and [Xylose_Pot_] is the potential xylose (g/L) present in the media used.

## Supplementary information

**Additional file 1.** Fermentation of ER-X, PE-2-X, CAT-1-X and CA11-X in in corn cob liquor supplemented with 100 g/L glucose at 40 °C; Growth profiles of ER-X, CAT-1-X and CA11-X and corresponding type strains in YPD; Stability evaluation of the integration at the δ-sequences; Fermentation of corn cob liquor with ER-X-2P strain and 2 cycles of cell recycling; Enzymatic activities; Plasmids and primers used for subcloning steps.

## Data Availability

The data sets used and/or analyzed in this study are available from the corresponding author upon reasonable request.

## Abbreviations

CBP: Consolidated bioprocessing
XOS: Xylooligosaccharides
S_0_: Severity
LSR: Liquid–solid ratio
HMF: Hydroxymethylfurfural
BGL1: β-Glucosidase 1
XYLA: β-Xylosidase A
XYN: Endoxylanase II
GOS: Glucooligosaccharides
XR: Xylose reductase
XDH: Xylitol dehydrogenase
XI: Xylose isomerase
SSCF: Simultaneous saccharification and co-fermentation
SSF: Simultaneous saccharification and fermentation
DCW: Dry cell weight
HPLC: High-performance liquid chromatography
