# Supplementary material for: Consolidated bioprocessing of corn cob-derived hemicellulose: engineered industrial Saccharomyces cerevisiae as efficient whole cell biocatalysts
Source: Biotechnol Biofuels. 2020 Aug 8;13:138. doi: 10.1186/s13068-020-01780-2 (PMC7414751; doi:10.1186/s13068-020-01780-2)
Supplement: Supplementary file 1 — Additional file 1. Fermentation of ER-X, PE-2-X, CAT-1-X and CA11-X in in corn cob liquor supplemented with 100 g/L glucose at 40 °C; Growth profiles of ER-X, CAT-1-X and CA11-X and corresponding type strains in YPD; Stability evaluation of the integration at the δ-sequences; Fermentation of corn cob liquor with ER-X-2P strain and 2 cycles of cell recycling; Enzymatic activities; Plasmids and primers used for subcloning steps. [file 13068_2020_1780_MOESM1_ESM.pdf]

Consolidated bioprocessing of corn cob-derived hemicellulose: engineered industrial *Saccharomyces cerevisiae* as efficient whole cell biocatalysts

Joana T. Cunha[1*], Aloia Romaní[1], Kentaro Inokuma[2], Björn Johansson[3], Tomohisa Hasunuma[2,4], Akihiko Kondo[2,4], Lucília Domingues[1]

[1]CEB – Centre of Biological Engineering, University of Minho, Campus Gualtar, Braga, Portugal

[2]Graduate School of Science, Technology and Innovation, Kobe University, 1-1 Rokkodai-cho, Nada-ku, Kobe, Hyogo 657-8501, Japan

[3]Center of Molecular and Environmental Biology (CBMA), University of Minho, Braga, Portugal

[4]Engineering Biology Research Center, Kobe University, 1-1 Rokkodai-cho, Nada-ku, Kobe, Hyogo 657-8501, Japan

*Corresponding author: jcunha@ceb.uminho.pt

(JTC) jcunha@ceb.uminho.pt (AR) aloia@ceb.uminho.pt (KI) kinokuma@port.kobe-u.ac.jp (BJ) bjorn_johansson@bio.uminho.pt (TH) hasunuma@port.kobe-u.ac.jp (AK) akondo@kobe-u.ac.jp (LD) luciliad@deb.uminho.pt

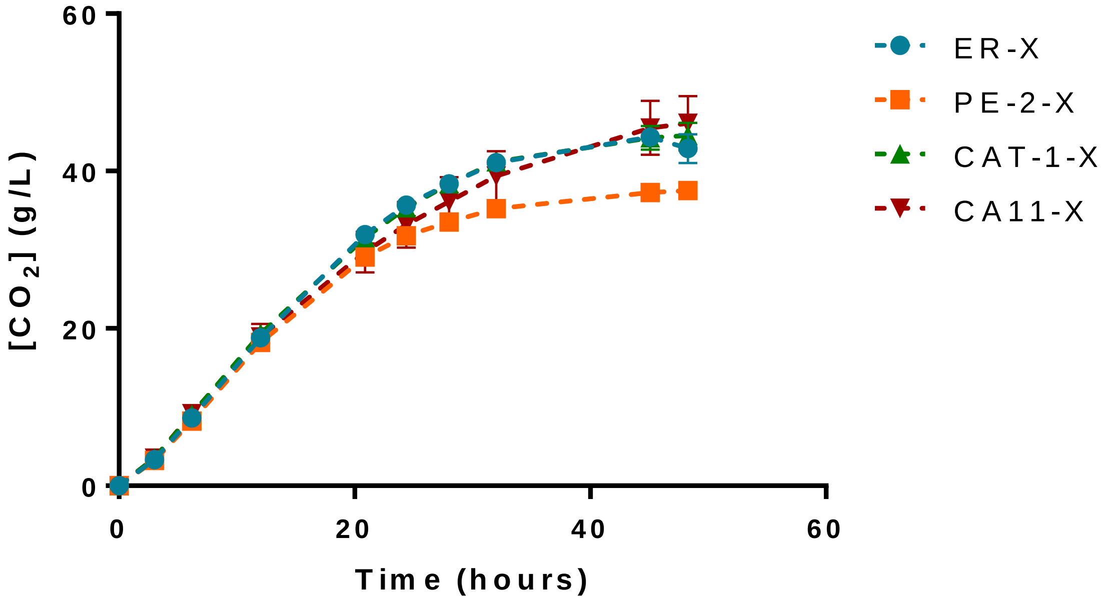

Fig. S1. Fermentation in corn cob liquor $29X_{Pot}$ (75 %) supplemented with 100 g/L glucose at 40 °C to evaluate the metabolic capacity of the different strains at higher temperature. Cells were inoculated at 10 g wet cells/L in flasks fitted with glycerol lock. The fermentation was performed at 40 °C and 150 rpm for 48 hours and was monitored by measuring the reduction of mass loss resulting from CO_2 production. Data represents the average ± SD from two biological replicates.

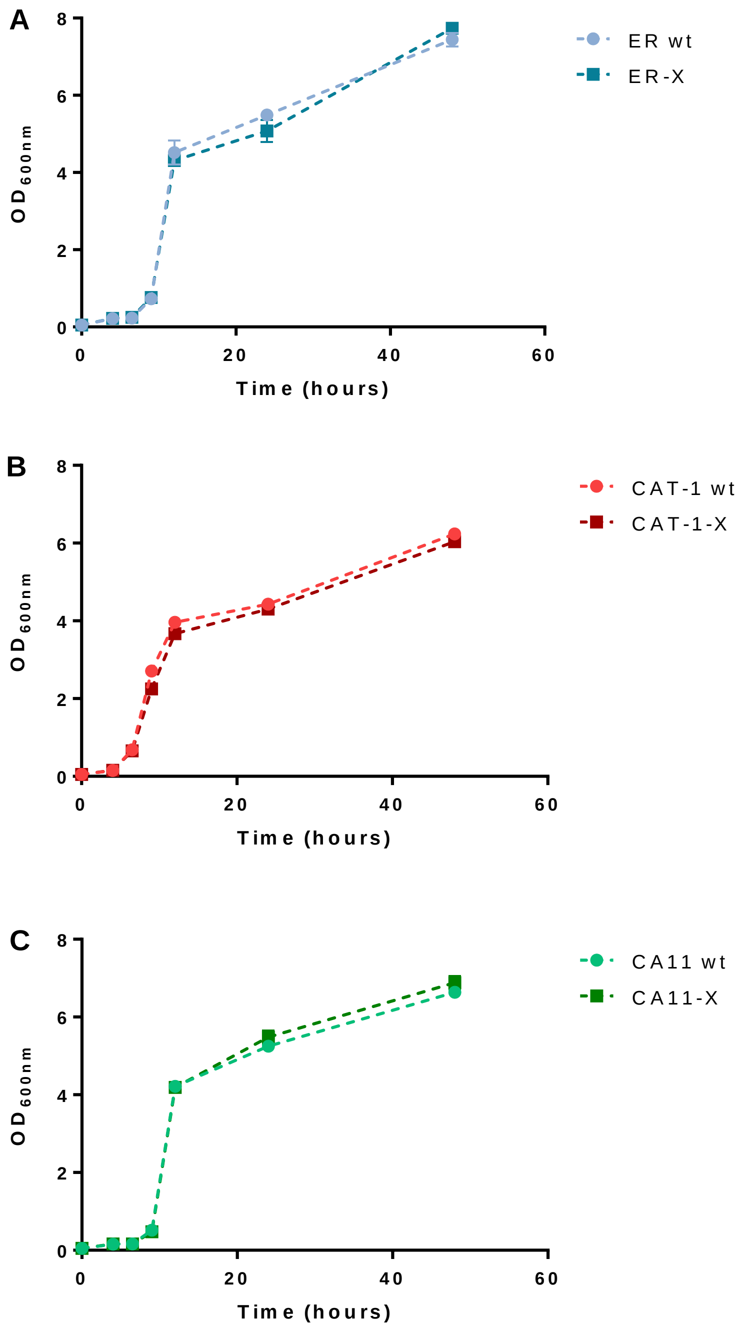

Fig. S2. Growth profiles of the wild type *S. cerevisiae* strains, ER, CAT-1 and CA11, and of the strains with cell surface display of hemicellulolytic enzymes, ER-X, CAT-1-X and CA11-X, in YPD. Cells were inoculated at an OD_{600nm} of 0.05 and grown for 48 hours at 30 °C and 200 rpm. Data represents the average ± SD from two biological replicates.

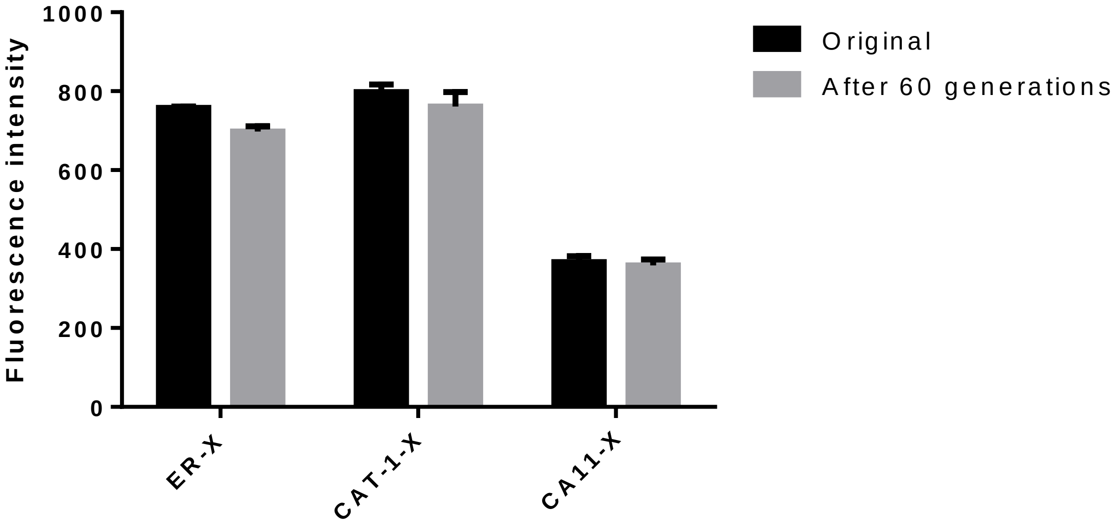

Fig. S3. Stability evaluation of the integration at the δ-sequences after growth for 60 generations. Cells were grown in YPD liquid medium for 60 generations. The fluorescence intensity of the cells before and after 60 generations (normalized to an OD_{600nm} of 0.2) was measured in a microplate fluorometer set at 480 nm (excitation) and 510 nm (emission). Data represents the average ± SD from two biological replicates.

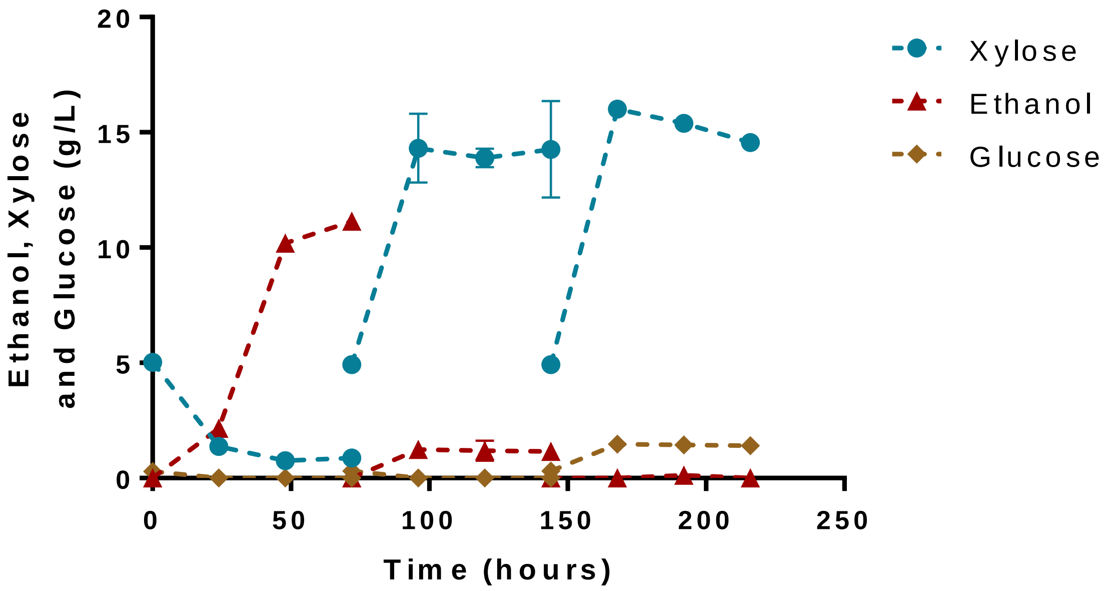

Fig. S4. Fermentation in corn cob liquor $32X_{Pot}$ with ER-X-2P strain and 2 cycles of cell recycling. Data represents the average ± SD from two biological replicates.

Table S1. β-glucosidase 1 (BGL1), β-xylosidase A (XYLA) and endoxylanase II (XYN) enzymatic activities.

Strain	BGL1 (U/g DCW)	XYLA (U/g DCW)	XYN (U/g DCW)	
			30 °C	40 °C
ER	39.1	87.8	139 ± 24	263 ± 2
PE-2	71.1	155	93.1 ± 2.2	162 ± 1
CAT-1	50.6	50.2	76.4 ± 3.7	140 ± 2
CA11	71.3	47.8	41.0 ± 8.0	57.9 ± 1.1

Table S2. Plasmids and primers used for subcloning steps in this study. Lower case sequences indicate addition of homologous regions for plasmid assembling.

**Primers**	**Sequence (5’->3’)**	**Aim**
pAll_fw	CGCATCAGGAAATTGTAAACG	Amplification of pIBG-SSA for construction of pI23-BGL1-kanMX; Amplification of pIU5-TeCBH1c-SSS for construction of pI5-CBH1-NatMX
pBGL1_CBH2_rv	CGTCAGGTGGCACTTTTC	Amplification of pIBG-SSA for construction of pI23-BGL1-kanMX
I23-BGL1_fw	aagtgccacctgacgACAGAGAAGGACAAGGCTGAAG	Amplification of intergenic region 23 from ER for construction of pI23-BGL1-kanMX
I23-BGL1_rv	cgacctgcagcgtacGACACAGGTGACAATAAAGTTTCC	
kanMX-BGL1_fw	GTACGCTGCAGGTCGACAAC	Amplification of *kanMX* resistance marker for construction of pI23-BGL1-kanMX
kanMX-BGL1_rv	caatttcctgatgcgATAGGCCACTAGTGGATCTGATATC	
I23_FW	ATAATGAGTTCCGAGTCTGTTGGTG	Confirm integration in intergenic region 23
I23_RV	CGAGATAAGGCATGGGGTTCTG	
pI5-CBH1_rv	CAGGTTGTGCTCACTGTATATAGTC	Amplification of pIU5-TeCBH1c-SSS for construction of pI5-CBH1-NatMX
Nat-pI5-CBH1_fw	agtgagcacaacctgGACATGGAGGCCCAGAATAC	Amplification of nat*MX* resistance marker for construction of pI5-CBH1-NatMX
Nat-pI5-CBH1_rv	caatttcctgatgcgCAGTATAGCGACCAGCATTC	
pI5-NatMX_fw	TGCTGGCGTTTTTCCATAG	Amplification of pI5-CBH1-NatMX for construction of pI5-XylA-NatMX
pI5-NatMX_rv	CAGCCTGAATGGCGAATG	
XylA_fw	tcgccattcaggctgCTTCGCTATTACGCCAGATTG	Amplification of pIK-BX-SSS for construction of pI5-XylA-NatMX
XylA_rv	ggaaaaacgccagcaCGAATTGGGTACCTTTGATTATG	
H-73	TCTCTCTTGCACCAGCCATT	Confirm integration in intergenic region I5
H-75	CGGAATCGCATCAGGTCTT	
pδW_fw	CTGAGAAATGGGTGAATGTTGAG	Amplification of pδW-EX-SSS for construction of pδW-XYN-kanMX
pδW_rv	CGGGGGATCCACTAGTTCTAG	
kanMX-UkG1_fw	ctagtggatcccccgGACACACAAAATATCCCTTCCTACTC	Amplification of kanMX-UkG1 cassette for construction of pδW-XYN-kanMX
kanMX-UkG1_rv	tcacccatttctcagGGTGTCGACAACCCTTAATATAACTTC	
pCRE_fw	CCATCTTGCACTTCAATAGCATATC	Amplification of pBF3060 for construction of pCRE-hyg6
pCRE_rv	GTATGAGTATTCAACATTTCCGTGTC	
hyg6_fw	gttgaatactcatacGACATGGAGGCCCAGAATAC	Amplification of *hyg6* resistance marker for construction of pCRE-hyg6
hyg6_rv	tgaagtgcaagatggCAGTATAGCGACCAGCATTCAC	
**Plasmids**	**Relevant features**	**Source**
pIBG-SSA	SED1p–SED1ss–*A. aculeatus* BGL1–SAG1a–SAG1t, *HIS3*	[1]
pIU5-TeCBH1c-SSS	SED1p–SED1ss–*T. emersonii* CBHI–SAG1a–SAG1t, *URA3*	[2]
pI5-CBH1-NatMX	SED1p–SED1ss–*T. emersonii* CBHI–SAG1a–SAG1t, *NatMX*	This work
pIK-BX-SSS	SED1p–SED1ss–*A. oryzae* XYLA–SED1a–SAG1t, *LYS2*	[3]
pδW-EX-SSS	SED1p–SED1ss–*T. reesei* XYNII–SED1a–SAG1t, *TRP1*, δ-integration	[3]
pBF3060	CRE recombinase, *URA3*	[4]

References

1. Inokuma K, Hasunuma T, Kondo, A. Efficient yeast cell-surface display of exo- and endo-cellulase using the SED1 anchoring region and its original promoter. Biotechnol Biofuels. 2014;7:8.

2. Liu Z, Inokuma K, Ho S-H, Haan Rd, Hasunuma T, van Zyl WH, et al. Combined cell-surface display- and secretion-based strategies for production of cellulosic ethanol with *Saccharomyces cerevisiae.* Biotechnol Biofuels. 2015;8:162.

3. Guirimand G, Inokuma K, Bamba T, Matsuda M, Morita K, Sasaki K, et al. Cell-surface display technology and metabolic engineering of *Saccharomyces cerevisiae* for enhancing xylitol production from woody biomass. Green Chem. 2019;21:1795-808.

4. Fang F, Salmon K, Shen MWY, Aeling KA, Ito E, Irwin B, et al. A vector set for systematic metabolic engineering in *Saccharomyces cerevisiae.* Yeast. 2011;28:123-36.
